# Effect of a bacteriocin-producing *Streptococcus salivarius* on the pathogen *Fusobacterium nucleatum* in a model of the human distal colon

**DOI:** 10.1080/19490976.2022.2100203

**Published:** 2022-07-25

**Authors:** Garreth W. Lawrence, Niamh McCarthy, Calum J. Walsh, Tais M. Kunyoshi, Elaine M. Lawton, Paula M. O’Connor, Máire Begley, Paul D. Cotter, Caitriona M. Guinane

**Affiliations:** a Department of Biological Sciences, Munster Technological University, Cork, Ireland; b Food Biosciences, Teagasc Food Research Centre Moorepark, Cork, Ireland; c VistaMilk SFI Research Centre, Moorepark, Fermoy, Cork, Ireland

**Keywords:** *Fusobacterium nucleatum*, *Streptococcus salivarius*, bacteriocins, biotherapeutics, probiotics, colorectal cancer, colon model

## Abstract

The gut microbiome is a vast reservoir of microbes, some of which produce antimicrobial peptides called bacteriocins that may inhibit specific bacteria associated with disease. *Fusobacterium nucleatum* is an emerging human bacterial pathogen associated with gastrointestinal diseases including colorectal cancer (CRC). In this study, fecal samples of healthy donors were screened for potential bacteriocin-producing probiotics with antimicrobial activity against *F. nucleatum*. A novel isolate, designated as *Streptococcus salivarius* DPC6993 demonstrated a narrow-spectrum of antimicrobial activity against *F. nucleatum in vitro. In silico* analysis of the *S. salivarius* DPC6993 genome revealed the presence of genes involved in the production of the bacteriocins salivaricin A5 and salivaricin B. After 6 h in a colon fermentation model, there was a significant drop in the number of *F. nucleatum* in samples that had been simultaneously inoculated with *S. salivarius* DPC6993 + *F. nucleatum* DSM15643 compared to those inoculated with *F. nucleatum* DSM15643 alone (mean ± SD: 9243.3 ± 3408.4 vs 29688.9 ± 4993.9 copies/μl). Furthermore, 16S rRNA amplicon analysis revealed a significant difference in the mean relative abundances of *Fusobacterium* between samples inoculated with both *S. salivarius* DPC6993 and *F. nucleatum* DSM15643 (0.05%) and *F. nucleatum* DSM15643 only (0.32%). Diversity analysis indicated minimal impact exerted by *S. salivarius* DPC6993 on the surrounding microbiota. Overall, this study highlights the ability of a natural gut bacterium to target a bacterial pathogen associated with CRC. The specific targeting of CRC-associated pathogens by biotherapeutics may ultimately reduce the risk of CRC development and positively impact CRC outcomes.

## Introduction

The gastrointestinal tract (GI) harbors trillions of diverse microbes, including candidate biotherapeutic bacteria such as probiotics. Probiotic bacteria are defined as “live microorganisms that, when administered in adequate amounts, confer a health benefit on the host”.^1^ Probiotic bacteria can exert health benefits on its host through several mechanisms, including targeting pathogenic microbes via antimicrobial production.^2^ Therefore, the GI tract has been regarded as a reservoir for novel antimicrobials, and extensive research has been focused on screening bacterial gut isolates for antimicrobial compounds such as bacteriocins.^3,4^ Bacteriocins are antimicrobial peptides produced by specific bacteria, which exhibit potent activity against other bacteria.^5^ It should be noted that the *in vitro* inhibitory activity of bacteriocins against a particular target does not necessarily translate to the gut environment. *Ex vivo* models provide a convenient means of bridging the gap between *in vitro* and *in vivo* investigations to assess the impact of different modulators on the gut microbiota.^6,7^ Indeed, *ex vivo* models of the colon have been used on a number of occasions to evaluate the impact of antibiotics^8^ and bacteriocins^9,10^ on intestinal microbial communities.

The role of the gut microbiota in colorectal cancer (CRC) has been the focus of ever increasing interest and a number of bacterial species have recently been associated with the disease.^11,12^ *Fusobacterium nucleatum* is an emerging pathogen shown to be associated with many GI diseases but specifically with CRC, where high abundances of *Fusobacterium* and *F. nucleatum* have been identified in colon cancer samples by transcriptomic and metagenomic profiling, in comparison to healthy controls.^13–19^ Indeed, there is now increasing evidence to suggest that the association between *F. nucleatum* and CRC reflects causation rather than correlation,^20,21^ thus making *F. nucleatum* a potential therapeutic target for prevention of this disease. Targeting specific species associated with CRC, while exerting minimal impact on the surrounding microbiota, is an attractive proposition and, thus, narrow-spectrum antimicrobials, such as bacteriocins, are particularly relevant.

The genus *Streptococcus* comprises some health-promoting members including strains of the species *Streptococcus salivarius*. These health-promoting attributes have been identified in the *S. salivarius* M18^22^ strain and the commercially available bacteriocin-producing *S. salivarius* K12 strain. *S. salivarius* K12 exerts narrow-spectrum antimicrobial activity against *Streptococcus pyogenes*,^23^ an oral pathogen associated with many oral pathologies including pharyngitis. *S. salivarius* K12 has shown promise for the treatment of oral streptococcal diseases in numerous clinical trials^24^ and may also have health benefits at other body sites.^25^ On the basis of this evidence, there is merit in investigating the application of other *S. salivarius* strains for health promoting purposes. Here, we screened fecal samples of healthy donors and isolated a potential bacteriocin-producing probiotic designated *S. salivarius* DPC6993 with antimicrobial activity against *F. nucleatum*. Then, we investigated the impact of *S. salivarius* DPC6993 on *F. nucleatum* in an *ex vivo* model of the human colon.

## Results

### Screening and isolation of bacteriocin-producing bacteria

A large-scale screen for antimicrobial activity against *F. nucleatum* was performed using agar-based deferred antagonism assays. Screening of over 16,000 colonies from 17 fecal samples from a cohort of healthy donors resulted in the detection of 56 colonies exhibiting antimicrobial activity against *F. nucleatum* DSM15643. This number was subsequently reduced to five isolates following repeat overlay assays (to ensure the retention of this initial phenotype) and well assays with buffered and unbuffered cell-free supernatant (in order to rule out inhibitory activity due to organic acid production). 16S rRNA gene sequencing established that all of the five isolates were speciated as *S. salivarius*. A single isolate was taken forward for further characterization and designated *S. salivarius* DPC6993.

### Antimicrobial-producing capacity of *S.* *salivarius* DPC6993

**Figure 1.**
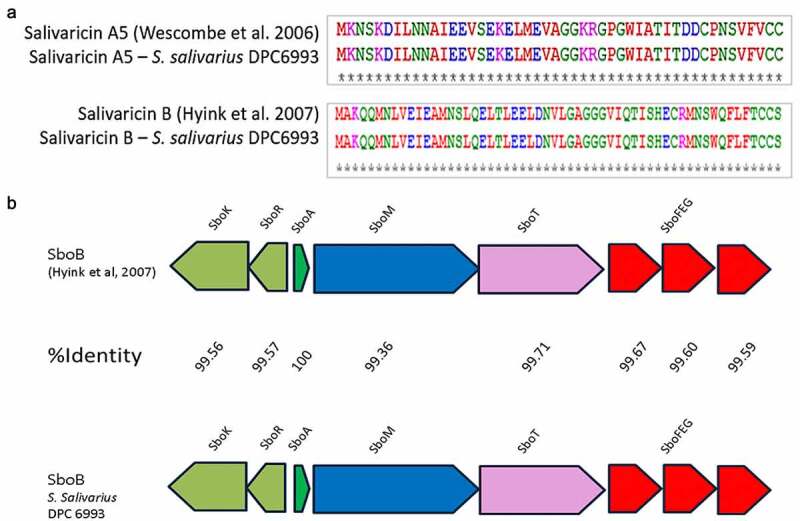
**Sequence alignments of salivaricin A5 from *S. salivarius* H21f^42^ and salivaricin B from *S. salivarius* K12^43^ with *S. salivarius* DPC6993**. The structural peptides found in the *S. salivarius* DPC6993 showed 100% amino acid identity to previously characterized bacteriocins (a). Comparison of genetic structure of previously characterized salivaricin B (SboB)^30^ operon (top) present in *S. salivarius* K12 and genetic structure of salivaricin B (SboB) operon (bottom) found in *S. salivarius* DPC6993. The percentage amino acid identities of the genes within the bacteriocin operons are given and were determined by performing multiple sequence alignments using Clustal Omega (b).

**Figure 2.**
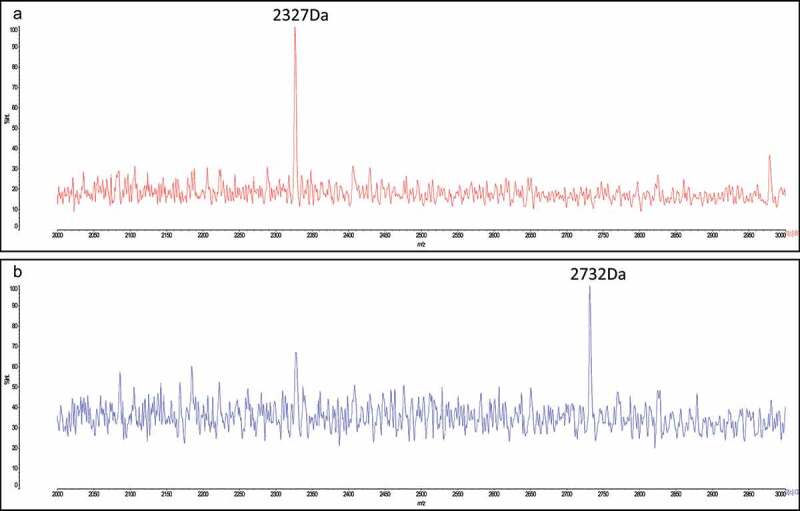
**Colony mass spectrometry on *S. salvarius* DPC6993**. Colony mass spectrometry revealed a 2327 Da mass (a), similar to the mass of a previously characterized bacteriocin Salivaricin A5 secreted by *S. salivarius* H21f and a 2732 Da mass (b), similar to the mass of previously characterized bacteriocin salivaricin B (SboB) secreted by *S. salivarius* K12.

Following genome sequencing of *S. salivarius* DPC6993, the draft genome was screened for the presence of genes that encode putative antimicrobial peptides, specifically bacteriocins, using the software BAGEL3, an automated bacteriocin mining tool.^26^ Analysis revealed two putative bacteriocin clusters corresponding to salivaricin B^27^ and salivaricin A5^28^ bacteriocins, confirmed by multiple sequence alignments on Clustal Omega.^29^ The sequences of the predicted structural peptides of the two clusters had 100% amino acid identity with these salivaricins (Figure 1a /1B). Colony mass spectrometry analysis of *S. salivarius* DPC6993 revealed the presence of 2327 Da (Figure 2a) and 2732 Da peptide mass signals (Figure 2b), corresponding to the mass of salivaricin A5 and salivaricin B, respectively.

### Inhibitory spectrum of *S.* *salivarius* DPC6993

**Figure 3.**
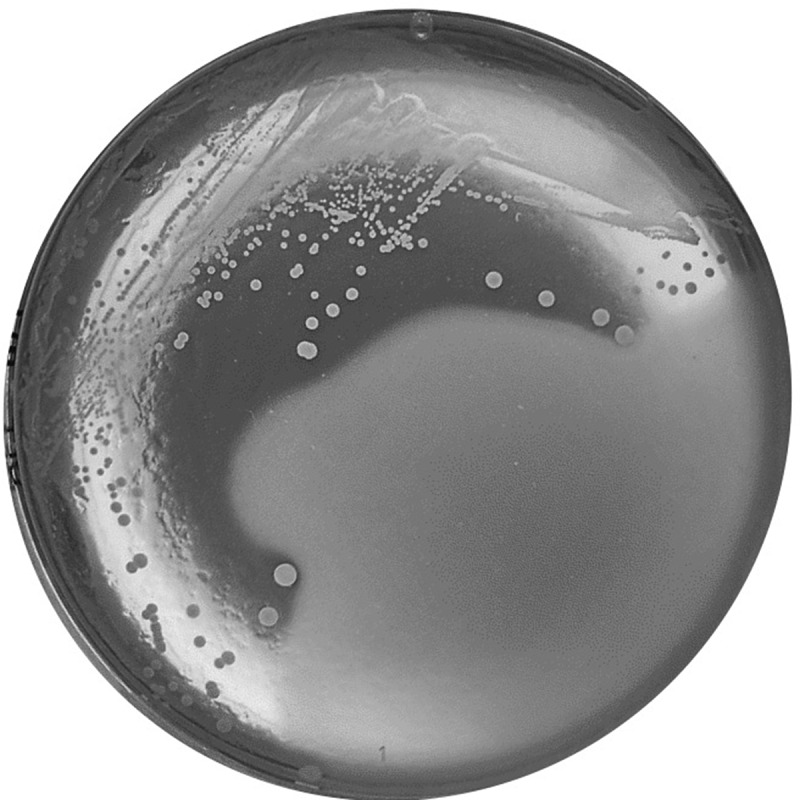
**Inhibition of *F. nucleatum* DSM15643 by *S. salivarius* DPC6993 demonstrated by a deferred antagonism assay**. Fully grown cultures of *S. salivarius* DPC6993 on brain heart infusion (BHI) agar were overlayed with fastidious anaerobic agar (1.5% w/v) seeded with *F. nucleatum* DSM15643 (7.5%) and incubated for 24 h anaerobically at 37°C.

Initially, *S. salivarius* DPC6993 demonstrated antimicrobial activity against *F. nucleatum* DSM15643 (Figure 3) and *Lactobacillus delbrueckii* subsp. *bulgaricus* DPC5383 producing zones of 5–8 mm and 8–10 mm, respectively. The spectrum of activity of *S. salivarius* DPC6993 against a panel of bacterial strains representing those typically found in the mammalian gastrointestinal and urogenital tracts was further tested **(Table S2)**. A relatively narrow spectrum of inhibition was evident with activity observed only against the seven *F. nucleatum* strains including DSM15643, DSM19507, DSM19508, DSM19679, D11(BEI) and CTI-01(BEI), one other *Fusobacterium* species *F. periodinticum* DSM19545 and seven other gut associated bacteria including *Lactobacillus delbrueckii* subsp. *bulgaricus* DPC5383, *Bifidobacterium breve* DPC6325, *Bifidobacterium longum* DPC6316, *Clostridioides difficile* DPC6507 and DPC6510, and *Streptococcus mutans* APC1076 **(Table S2)**.

### Impact of the bacteriocin-producing *S.* *salivarius* DPC6993 on *F.* *nucleatum i*n a model colonic environment

**Figure 4.**
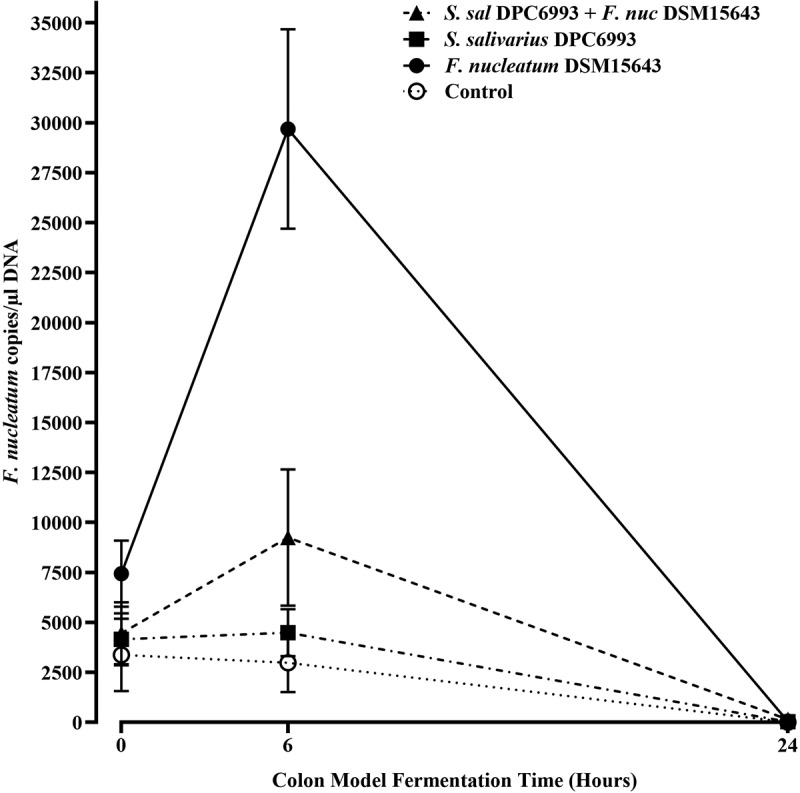
**Quantification of *F. nucleatum* in colon model wells as determined by qPCR analysis at 0, 6 and 24 h**. A significant difference (*p* = .00034) in *F. nucleatum* numbers was observed in colon model wells that had been simultaneously inoculated with *S. salivarius* DPC6993 compared to those inoculated with *F. nucleatum* DSM15643 alone. Mean *F. nucleatum* copy numbers and SDs for each condition were derived from three colon model wells at each timepoint.

To determine the impact of the bacteriocin-producer *S. salivarius* DPC6993 on *F. nucleatum* DSM15643 numbers in a simulated colon environment, the two strains were inoculated into such an environment at 10^9^ and 10^6^ CFU/ml, respectively. Quantification of *F. nucleatum* was performed by real time-quantitative polymerase chain reaction (RT-qPCR) on DNA extracted from all colon model wells at time points T0, T6 and T24 post-inoculation. Four variations were studied: *S. salivarius* DPC6993 with *F. nucleatum* DSM15643; *S. salivarius* DPC6993 only; *F. nucleatum* DSM15643 only and controls (blank fecal slurry) (Figure 4). Studies were performed in triplicate for each condition and represented by the mean copy of *F. nucleatum* ± SD. The qPCR analysis revealed that after 6 h (T6), there was a significant drop in the numbers of *F. nucleatum* in samples that had been simultaneously inoculated with *S. salivarius* DPC6993 + *F. nucleatum* DSM15643 compared to those inoculated with *F. nucleatum* DSM15643 alone (9243.3 ± 3408.4 vs 29688.9 ± 4993.9 Fn copies/μl, *p* = .00034) (Figure 4). At T0, there was a significant difference (*p* = .001) when comparing *F. nucleatum* numbers between colon model wells inoculated with *S. salivarius* DPC6993 and *F. nucleatum* DSM15643 vs inoculated with *F. nucleatum* DSM15643 only (4452.8 ± 1547.2 vs 7437.9 ± 1650.7 Fn copies/μl). The significant difference observed here may be due to the dominance of *S. salivarius* DPC6993 and rapid bacteriocin induction when exposed to *F. nucleatum*. When *F. nucleatum* DSM15643 only, was injected into the colon model, a fourfold increase was observed after 6 h (T6) (7437.9 ± 1650.7 to 29688.9 ± 4993.9 Fn copies/μl). However, when both *F. nucleatum* DSM15643 and *S. salivarius* DPC6993 strains were simultaneously injected into the colon model, just a 2.1-fold increase in *F. nucleatum* numbers was observed (4452.8 ± 1547.2 to 9243.3 ± 3408.4 Fn copies/μl). After 24 h (T24), *F. nucleatum* was depleted in all colon model wells. It was noted that *F. nucleatum* was also detected in wells inoculated with *S. salivarius* DPC6993 only and control wells at low levels across all timepoints, indicating low levels of *F. nucleatum* already naturally present in the standardized fecal inoculum **(Table S3)**.

### Impact of *S.* *salivarius* DPC6993 on fecal bacterial diversity

**Figure 5.**
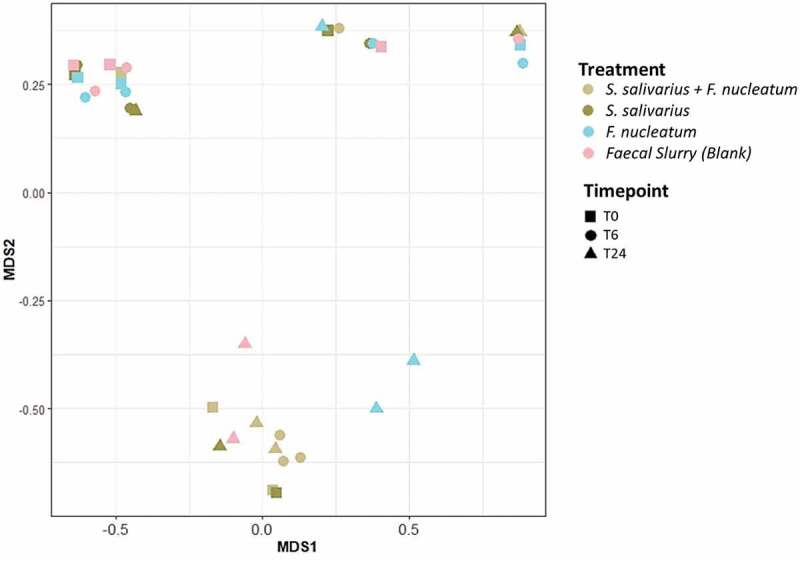
**Beta diversity analysis of colon model wells**. Beta diversity, represented by Bray–Curtis multidimensional scaling (MSD) analysis of fecal microbiota compositions in colon model wells inoculated with *S. salvarius* DPC6993 and *F. nucleatum* DSM15643, *S. salivarius* DPC6993, *F. nucleatum* DSM15643 and control wells at T0, T6 and T24.

The impact of introducing the bacteriocin producer on the overall diversity of bacterial populations in the colon was also investigated. To assess the impact on beta diversity, multidimensional scaling (MDS) plots were generated based on the Bray–Curtis Dissimilarity method. The MDS plot based on the calculated distance matrices shows no clustering by treatment or timepoint, indicating no difference in microbial diversity (Figure 5). The Adonis analysis was not significant when calculated for treatment (*p* = .237) or timepoint (*p* = .198).

Alpha diversity metrics were calculated to evaluate the diversity within colon model wells. There were no significant differences in the number of observed genera within timepoints between colon model treatments and controls, except for wells inoculated with *F. nucleatum* DSM15643 vs control at T6 (*p* = .03) (Table 1**; Figure S1)**. Shannon and Simpson diversity, reflective of richness and evenness of genera were similar at T0 for all treatments and decreased at T6 and T24 in *S. salivarius* DPC6993 + *F. nucleatum* DSM15643 and *S. salivarius* DPC6993 wells (Table 1). Alpha diversity was significantly higher in *F. nucleatum* DSM15643 and control wells compared to *S. salivarius* DPC6993 + *F. nucleatum* DSM15643 and *S. salivarius* DPC6993 wells as determined by Simpson and Shannon indices at T6 and T24 (Table 1**; Figure S1)**. However, there were no significant differences in *S. salivarius* DPC6993 + *F. nucleatum* DSM15643 (Shannon *p* = .84; Simpson *p* = .74) and *S. salivarius* DPC6993 (Shannon *p* = .58; Simpson *p* = .48) wells at T6 compared to T24 indicating that the alpha diversity remained stable and that the initial differences were driven by the dominance of *S. salivarius* DPC6993 cells (Table 1**; Figure S1)**. *F nucleatum* DSM15643 and control wells diversity measures remained similar over the 24 h period. Both Shannon and Simpson metrics for all wells at T24 were similar to T6 (Table 1**; Figure S1)**.

**Table 1.** Estimates of alpha diversity for colon model treatments at each timepoint.

Colon model treatment	Timepoint	Simpsons Diversity Index	Shannon Index	Number of observed genera
	T0	0.86 ± 0.07	2.88 ± 0.40	117 ± 15
*S. salivarius* DPC6993 + *F. nucleatum* DSM15643	T6	0.48 ± 0.06	1.41 ± 0.16	79 ± 5
	T24	0.50 ± 0.02	1.43 ± 0.03	77 ± 17
	T0	0.87 ± 0.01	2.89 ± 0.04	123 ± 17
*S. salivarius* DPC6993	T6	0.51 ± 0.04	1.50 ± 0.13	88 ± 38
	T24	0.54 ± 0.04	1.56 ± 0.11	88 ± 9
	T0	0.94 ± 0.001	3.35 ± 0.02	103 ± 14
*F. nucleatum* DSM15643	T6	0.81 ± 0.02	2.22 ± 0.11	103 ± 14
	T24	0.85 ± 0.04	2.58 ± 0.17	87 ± 19
	T0	0.86 ± 0.07	2.76 ± 0.55	102 ± 22
Control	T6	0.82 ± 0.02	2.33 ± 0.15	90 ± 10
	T24	0.84 ± 0.00	2.48 ± 0.05	80 ± 25

### Impact of *S.* *salivarius* DPC6993on intestinal bacterial populations

**Figure 6.**
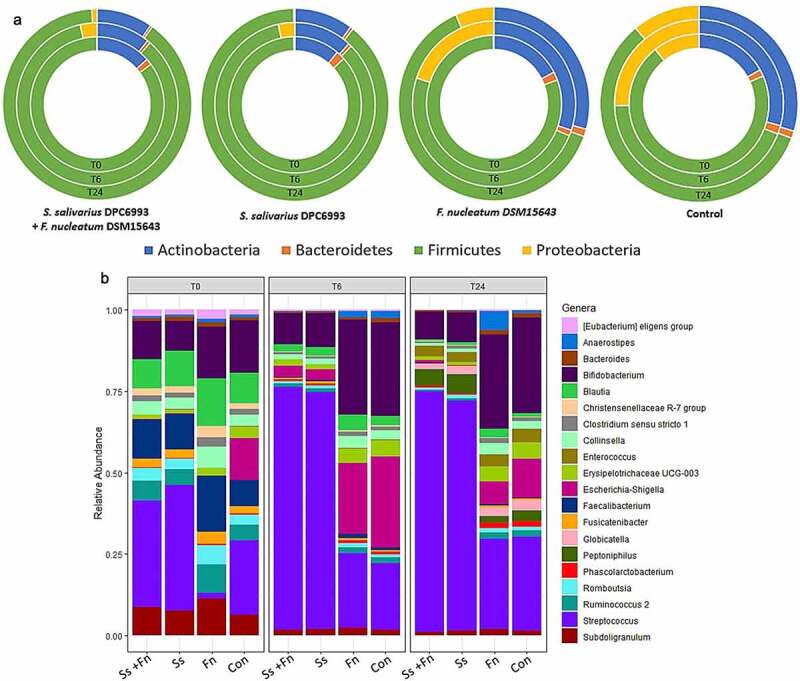
**Relative abundances of phylum** (a) **and genera** (b) **in colon model wells with respect to treatment and timepoint**. The dominant taxa are labeled. Relative abundance are represented by the mean of three colon model wells. Ss+Fn, *S. salivarius* DPC6993* + F. nucleatum* DSM15643; Ss, *S. salivarius* DPC6993; Fn, *F. nucleatum* DSM15643; Con, Control.

To determine the impact of *S. salivarius* DPC6993 on the composition of the model colonic microbiota, metagenomic DNA was extracted from the model colon samples at T0, T6 and T24 for all treatments and subjected to 16S rRNA sequencing to determine relative abundance of microbial communities. At the phylum level, the most dominant phyla in all colon model treatments across all timepoints were Firmicutes and Actinobacteria with smaller proportions of Proteobacteria, Bacteroidetes and Fusobacteria (Figure 6a). At T6, all colon model wells show an increase in Proteobacteria with a decrease in Firmicutes populations, however a larger increase in Proteobacteria was found in *F. nucleatum* DSM15643 and control wells relative to the *S. salivarius* DPC6993 + *F. nucleatum* DSM15643 and *S. salivarius* DPC6993 wells (2.7% and 3.3% vs 24.5% and 25.4%, respectively), which may suggest *S. salivarius*’ ability to control Proteobacteria taxa. At T24, proportions of the main phyla remain similar to T6 in all treatments, with abundances of Proteobacteria subsiding (Figure 6a**; Table S4)**.

At the genus level, the most dominant genus in the *S. salivarius* DPC6993-treated wells was *Streptococcus* (Figure 6b). The next most abundant genera were consistent with those found in control samples. The relative abundance of *Streptococcus* increased from T0 (26.3% and 31.3%) to T6 (70.9% and 68.8%) in wells initially inoculated with *S. salivarius* DPC6993 + *F. nucleatum* DSM15643 and *S. salivarius* DPC6993, respectively. At T24, *Streptococcus* abundances remained similar to abundances observed at T6. In the *F. nucleatum* DSM15643-containing and control wells, *Streptococcus* abundances were detected at 1.2% and 19% at T0; 24.1% and 18.7% at T6; and 24.4% and 26.02% at T24. Other dominant genera at T0 in all colon model wells are *Bifidobacterium, Blautia, Faecalibacterium, Ruminococcus*, and *Subdoligranulum*, with a large proportion of reads assigned to *Escherichia-Shigella* (10.7%) in control wells relative to all other treatments. At T6, the dominant genera observed in *S. salivarius* DPC6993 + *F. nucleatum* DSM15643 and *S. salivarius* DPC6993 wells decrease as most reads are assigned to the rapid growth in *Streptococcus*. After 6 h, the *F. nucleatum* DSM15643 and control wells showed a relative increase in *Anaerostipes* (0.8–1.4% and 0.7–2%), *Bifidobacterium* (11.6–25.2% and 13.4–26.3%) and a decrease in *Blautia* (10.5–2.7% and 7.9–2.4%), *Faecalibacterium* (12.3–0.83% and 6.7–0.9%) and *Ruminococcus* (6.4–1.02% and 4.1–1.5%) relative to T0. An increase in *Enterococcus, Peptoniphilus* and *Globicatella* is observed in all treatments at T24 with relative abundances of other genera similar to T6. At T6, a larger increase in *Escherichia–Shigella* is observed in the *F. nucleatum* DSM15643 and control wells (0.13–24.97% and 10.7–25.3%) compared to *S. salivarius* DPC6993 + *F. nucleatum* DSM15643 and *S. salivarius* DPC6993 wells (0.08–3.47% and 0.23–3.22%) relative to T0. Reads assigned to *Escherichia-Shigella* at T24 in the *S. salivarius* DPC6993 + *F. nucleatum* DSM15643 and *S. salivarius* DPC6993 wells decrease relative to T6 (3.5–0.8% and 3.2–0.08%) and a similar pattern is observed in the *F. nucleatum* DSM15643 and control wells (24.97–6.1% and 25.3–10.8%) (Figure 6b**; Table S5)**.

**Figure 7.**
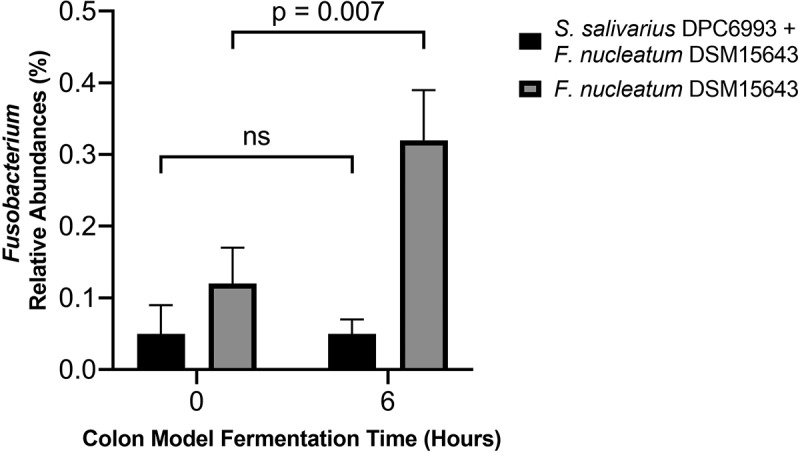
***Fusobacterium* relative abundances in colon model wells inoculated with *S. salivarius* DPC6993 and *F. nucleatum* DSM15643 vs colon model wells inoculated with *F. nucleatum* DSM15643 only**. At T24, no reads were assigned to *Fusobacterium.*

As the qPCR data demonstrated that *S. salivarius* DPC6993 may supress the growth of *F. nucleatum* within a simulated colon environment, we further hypothesized that there would be a decrease in taxa assigned to Fusobacteria and *Fusobacterium* in colon model wells inoculated simultaneously with *S. salivarius* DPC6993 and *F. nucleatum* DSM15643 compared to wells inoculated with *F. nucleatum* DSM15643 only. Fusobacteria abundances remained similar at T6 relative to T0 in the *S. salivarius* DPC6993 + *F. nucleatum* DSM15643 wells (0.05–0.05%) with a slight decrease in the *S. salivarius* DPC6993 wells (0.058–0.014%); however, abundances increased in the *F. nucleatum* DSM15643 wells (0.13–0.34%), suggesting *S. salivarius’* potential to suppress the growth of fusobacterial taxa (Figure 6a). No reads were assigned to Fusobacteria at T24 in all treatments or at T0 and T6 in the control wells. Figure 7 shows a significant difference in the relative abundances of *Fusobacterium* between colon model wells inoculated with both *S. salivarius* DPC6993 and *F. nucleatum* DSM15643 and *F. nucleatum* DSM15643 only. A 2.7-fold increase (0.12–0.32%) in *Fusobacterium* relative abundance is observed at T6 in wells inoculated with *F. nucleatum* DSM15643, compared to no change in *Fusobacterium* relative abundances at 0.05% when inoculated with *S. salivarius* DPC6993 at T6. At T24, no reads were assigned to *Fusobacterium* in colon model wells for either treatments.

## Discussion

The gut microbiota can be regarded as a reservoir of novel antimicrobials, including bacteriocins^3^ and the potential use of biotherapeutic bacteria, such as bacteriocin-producing probiotics, to target colorectal cancer-associated taxa has been proposed.^31^ Probiotics that produce bacteriocins have the potential to be utilized for the elimination of specific microbial pathogens, harnessing a narrow spectrum of activity, leaving the natural microbiota unharmed.^5,32^

The association of *F. nucleatum* and CRC is well established.^13^ Several studies highlight its overrepresentation in the tissue and stool samples of CRC patients,^14,17,33,34^ therefore identifying *F. nucleatum* as a therapeutic target. As *F. nucleatum* was first characterized as a dental pathogen, it is notable that a number of studies have shown the potential use of antimicrobial-producing bacteria^35–37^ and bacteria isolated from functional foods^38^ to inhibit it and other oral pathogens *in vitro*. The potential use of strains from the fermented milk, kefir, to inhibit *F. nucleatum in vitro* has also been noted.^39^ Recently, in a study which screened the athlete gut microbiome for antimicrobial-producers, two bacteriocin-producing strains of *Enterococcus faecalis* were isolated which demonstrated anti*-F*. nucleatum activity in lab media.^4^ Furthermore, probiotic interventional trials aimed at modifying the CRC-associated microbiota have caused a reduction in CRC-associated taxa including *Fusobacterium*^40,41^ ; however, there is limited evidence on targeting specific species associated with the disease within the CRC microbiome. These previous studies show that bacteria with anti-*F. nucleatum* activity *in vitro* exist and intervention trials show promise for probiotic bacteria to manipulate CRC-associated taxa. Moreover, targeting specific bacterial species associated with CRC development in the guts of healthy populations, while exerting minimal impact on the surrounding gut microbiota is desirable, and may contribute as a preventative strategy.^31^ The aim of the current study was to screen fecal samples of healthy donors for potential bacteriocin-producing isolates that demonstrated narrow-spectrum antimicrobial activity against *F. nucleatum*, and to subsequently confirm this activity in an *ex vivo* model of the human colon.

We first screened fecal samples of healthy donors for potential bacteriocin-producing isolates active against *F. nucleatum*. Following screening of 16,000 colonies and repeat antimicrobial assays, five candidate isolates were further studied. All five isolates were identified as *S. salivarius* by 16S rRNA gene sequencing. Again, repeat assays were performed which distinguished a single isolate with consistent antimicrobial activity against *F. nucleatum* and was subsequently given the strain designation *S. salivarius* DPC6993. Bioinformatic analysis and colony mass spectrometry confirmed the presence of two putative bacteriocin clusters on the genome of *S. salivarius* DPC6993, salivaricin A5^28^ and salivaricin B.^27^ Indeed, the species *S. salivarius* produces many other salivaricin variants and subtypes including salivaricin D, salivaricin E, and salivaricin 9.^42^ The use of *S. salivarius* as a beneficial bacterium has been widely studied and its potential health-promoting characteristics are especially evident by the commercial bacteriocin-producing probiotic strain *S. salivarius* K12.^24,43^ Several other strains of *S. salivarius* have been identified with antimicrobial activity against pathogenic bacteria, which is attributed to their bacteriocin arsenal. For example, *S. salivarius* strains HSISS4, YU10 or NU10 demonstrated antimicrobial activity *in vitro* against *Staphylococcus aureus*, enterococci, *Listeria monocytogenes, Micrococcus* luteus and related pathogenic species *Streptococcus pyogenes*, and *Streptococcus mutans*.^30,44^ Interestingly, a recent study found that, while salivaricin producing strains *S. salivarius* K12 and M18 did not inhibit *F. nucleatum in vitro*, both strains were able to co-aggregate with *F. nucle*atum and inhibit IL-6 and IL-8 secretion.^45^ However, we recently reported a novel nisin variant designated as nisin G produced by *S. salivarius* DPC6487 with *in vitro* antimicrobial activity against *F. nucleatum* and other streptococci.^46^ While these benefits show potential for targeting pathogenic bacteria associated with disease, several studies show additional potential benefits of *S. salivarius*, which may impact the gut, including anti-inflammatory properties^47^ and maintaining microbial homeostasis.^25^

This study showed that *S. salivarius* DPC6993 had a narrow spectrum of activity *in vitro*, against *Fusobacterium* species, including the CRC-associated *F. nucleatum*, therefore, confirming direct inhibition, having no activity against strains of the genera *Lactobacillus* (except for *Lactobacillus delbruekii* subsp. *bulgaricus), Enterococcus, Streptococcus, Bacteroides, Clostridium, Listeria, Salmonella, Staphylococcus, Porphymoronas, Atopobium* and *Gardnerella*. The identification of narrow-spectrum antimicrobials, is especially warranted, as the use of broad-spectrum antimicrobials creates a window for opportunistic pathogens to colonize the gut microbiome.^48^

As the inhibition of pathogenic bacteria does not necessarily translate beyond *in vitro* tests, we investigated the ability of *S. salivarius* DPC6993 to impact the growth of *F. nucleatum* in an *ex vivo* model of the human colon. This model simulates the complex and dynamic environment of the colon and, thus, provides an insight into the functionality of the antimicrobial within the human colon prior to human studies.^7^ An approach involving the preparation of a fecal standard^49^ was used to simulate the distal colon environment, and we used qPCR which is a much more sensitive approach to capture bacteria at low relative abundance and is preferable for the quantification of single species^50^ and 16S rRNA sequencing which are considered as complementary and reliable for microbiota profiling.^51^ Considering that *S. salivarius* DPC6993 was isolated from the human gut, it was postulated that it would be active against *F. nucleatum* in its natural environment. Indeed, this was evident as determined by qPCR, which showed a significant reduction in the growth of *F. nucleatum* when *F. nucleatum* DSM15643 was inoculated simultaneously with *S. salivarius* DPC6993 into a simulated colon environment compared to when inoculated individually. Further to this, it is evident from the 16S rRNA compositional analysis that the CRC-associated genus *Fusobacterium* is less abundant in colon model wells inoculated with *S. salivarius* DPC6993 and *F. nucleatum* DSM15643 relative to wells inoculated with *F. nucleatum* DSM15643 only.

The 16S rRNA compositional analysis revealed changes in the taxonomic profiles by addition of high numbers of *S. salivarius* DPC6993 cells. However, the presence of dominant fecal genera in control wells was also observed in *S. salivarius* DPC6993-treated wells at lower relative abundances across each timepoint, indicating dominance of *S. salivarius* rather than negatively impacting the surrounding microbiota. Notably, *Streptococcus* rapidly increased in *S. salivarius* DPC6993-treated wells to become the most dominant taxa compared to controls after 6 h and remained constant after 24 h. This indicates *S. salivarius*’ ability, to not only survive but to flourish in the harsh conditions of human colon environment. Surviving passage through the GI tract and remaining functional is a desirable characteristic of candidate probiotics.^52^

We also investigated how the presence of the bacteriocin-producing *S. salivarius* DPC6993 impacts on the overall diversity and richness in the colon model. Analysis of beta diversity by multidimensional scaling showed no clustering of datapoints by treatment or timepoint indicating no dramatic change in microbial composition. Alpha diversity reduced in the *S. salivarius* DPC6993-treated wells after 6 h fermentation; however, diversity did not change from 6 to 24 h, indicating that the initial differences were driven by the dominance of *S. salivarius* DPC6993 cells. Furthermore, there were no significant differences in the number of observed genera between colon model treatments and controls after 24 h fermentation, indicating the microbial richness remained stable. Overall, these results show that the bacteriocin-producing *S. salivarius* DPC6993 suppresses the growth of *F. nucleatum* in an *ex vivo* model of the human colon and exerts minimal impact on the overall diversity of the surrounding microbiota.

## Conclusion

This study used an *ex vivo* model of the human colon to investigate, for the first time, the impact of the bacteriocin-producing *S. salivarius* DPC6993 on *F. nucleatum*, a gut pathogen associated with CRC. Results indicate that *S. salivarius* DPC6993 suppresses the growth of the CRC-associated bacteria within the gut environment and exerts minimal impact on the surrounding gut microbiota. This study is an important finding prior to *in vivo* analysis to evaluate the potential use of such bacteriocin-producing strains as biotherapeutics for suppressing the growth of *F. nucleatum* in the human gut, ultimately reducing the risk of CRC development and positively impacting CRC outcomes.

## Materials and methods

### Bacterial strains and cultivation media

*S. salivarius* DPC6993 was cultivated under anaerobic conditions at 37°C in brain heart infusion (BHI, Difco Laboratories, Detroit, MI, USA) broth and medium. Agar of concentration 1.5% w/v was added for agar plates. *F. nucleatum* DSM15643 was the target organism used in this study. *F. nucleatum* DSM15643 was cultivated under anaerobic conditions at 37°C on fastidious anaerobe agar (FAA; Lab M, Lancashire, UK) supplemented with 7% defibrinated horse blood (Cruinn, Dublin, Ireland) and Wilkin–Chalgren Broth (WCB; Oxoid, Hampshire, UK). Both strains were grown anaerobically using anaerobic jars, Anaerocult A gas packs (Merck, Darmstadt, Germany) and a Don Whitley Anaerobic workstation (nitrogen 85%, carbon dioxide 5%, hydrogen 10%). All strains used in this study were grown at 37°C. For a full list of bacteria and their culture conditions used in the antimicrobial spectrum analysis, see **Table S1**.

### Large-scale screen for antimicrobial producers

Fecal samples used in a previous study,^53^ which were obtained from a cohort of healthy donors, were used in this study to screen for potential bacteriocin-producing strains active against *F. nucleatum* DSM15643. These were stored in a BSL-2 laboratory and maintained at −80°C in Teagasc Food Research Center, Moorepark, Fermoy, Ireland. Prior to screening, the samples were removed from storage and defrosted at 37°C in an anaerobic chamber (nitrogen 85%, carbon dioxide 5%, hydrogen 10%, Don Whitley Anaerobic Workstation). Under strict anaerobic conditions, 1 g of fecal matter was serially diluted in sterile phosphate buffered saline (PBS; Sigma Aldrich, Co. Wicklow, Ireland). 100 µl of each dilution was spread on fastidious anaerobe agar (FAA) and grown overnight anaerobically at 37°C. Resulting colonies were overlayed with ~10 ml of FAA containing a 7.5% inoculum of the *F. nucleatum* DSM15643 and the plates were incubated for a further 18–24 h and subsequently examined for zones of inhibition. Colonies producing distinct zones of inhibition were sub-cultured onto fresh FAA plates and subsequently stocked in 80% glycerol and stored at −20°C and −80°C for further analysis. Stocked potential bacteriocin-producers were shortlisted by repeat deferred antagonism assays as described and also agar well diffusion assays.^54^ For the well assays, molten agar (~45–50°C) was seeded with the *F. nucleatum* DSM15643 at a seed volume of 3.75%. The inoculated medium was immediately poured into sterile petri dishes and allowed to solidify and dry under anaerobic conditions. Wells of uniform diameter (5.5 mm) were bored in the agar. Aliquots of 50 µl of cell-free supernatant (CFS) from an overnight culture of potential bacteriocin-producers were dispensed into the wells and the plates were incubated overnight for the conditions of the indicator (37°C, anaerobic). pH neutralization of the CFS of the bacteriocin producers was performed by taking 10 ml of fully grown, overnight culture and centrifuging at 4600 rpm for 30 min, the supernatant was then removed and centrifuged again at 4600 rpm for 15 min. The resulting supernatant was then pH adjusted with 1 M NaCl to a pH of ~7.50 µl aliquots of this were dispensed into wells for well diffusion assays as described above. Following incubation, the plates were examined for zones of inhibition around the wells.

### Speciation of bacteriocin-producing isolates

Genomic DNA of isolates which produced clear zones of inhibition was extracted from culture cell pellets using the GenElute™ Bacterial Genomic DNA Kit (Sigma-Aldrich; Co. Wicklow, Ireland). Extraction of DNA was confirmed by agarose gel electrophoresis and subsequently the 16S rRNA gene was amplified using the following 16S eubacterial primers CO1; 5’-AGTTTGATCCTCCTGGCTCAG-3’ and CO2; 5’-TACCTTGTTACGACTT-3’.^55^ The DNA was amplified with Invitrogen Platinum PCR Supermix (ThermoFisher Scientific, Dublin, Ireland) and PCR reactions performed on the Applied Biosystems 2720 Thermocycler (ThermoFisher Scientific, Dublin, Ireland). The amplification cycle used was as follows: 94°C for 2 min, and 30 cycles of the following: 94°C for 30 s, 50°C for 30 s and 72°C for 1.5 min. The purity and quantity of DNA present was checked on the NanoDrop 1000 (ThermoFisher Scientific, Dublin, Ireland) and the PCR product was then purified using the QIAquick™ PCR Purification Kit (Qiagen; Manchester, UK). The complete sequence of the 16S rRNA gene was determined by Sanger sequencing (Beckman Coulter, Essex, UK). The species was putatively identified by comparing the resulting sequence with deposited species in the NCBI database (http://blast.ncbi.nlm.nih.gov/Blast.cgi) with a high percentage nucleotide identity (>98%).

### *Streptococcus salivarius* DPC6993 whole genome sequencing

The purity and concentration of the *S. salivarius* DPC6993 genomic DNA preparation was confirmed using the NanoDrop 1000 (ThermoFisher Scientific, Dublin, Ireland) and Qubit® 2.0 Fluorometer (ThermoFisher Scientific, Dublin, Ireland) according to the respective manufacturer’s protocols. To purify the DNA before beginning the library preparation the Power Clean DNA Clean-up Kit (MO-BIO laboratories; Carlsbad, CA, USA) was used. Genomic libraries were prepared using the Nextera XT Library Preparation kit (Illumina inc., San Diego, CA, USA). Whole genome sequencing was performed using the MiSeq v3 600 cycles Paired Ends kit on the Illumina MiSeq platform (Illumina inc., San Diego, CA, USA) at the Teagasc Food Research Center, Moorepark, Fermoy. The resulting reads were quality checked using FastQC^56^ and BBDuk was used to remove sequencing adapters and PhiX reads and perform quality trimming (https://sourceforge.net/projects/bbmap/). The paired end reads were assembled into contigs and scaffolds using the SPAdes Genome Assembler.^57^ Open reading frames of the draft genome were predicted using Prodigal^58^ and complementary gene calling and automated annotation was completed using the RAST annotation server.^59^ BAGEL3 software, an automated bacteriocin mining tool, was used to detect the presence of any putative bacteriocin operons.^26^ Manual analysis of the genome was then subsequently performed using the ARTEMIS genome browser.^60^ Manual annotation of the genes potentially involved and surrounding the putative bacteriocin operons was completed by using the BLASTp algorithm and the non-redundant database provided by the NCBI^61^(http://blast.ncbi.nlm.nih.gov).

### Colony mass spectrometry

Fully grown colonies of *S. salivarius* DPC6993 were mixed with 50 µl 2-propanol 0.1% TFA, vortexed three times and centrifuged at 14,000 rpm for 30 s. MALDI TOF mass spectrometry was performed on the cell supernatant using an Axima TOF^2^ MALDI-TOF mass spectrometer (Shimadzu Biotech, Manchester, UK). A 0.5 µl aliquot of matrix solution (α-cyano 4-hydroxy cinnamic acid, 10 mg/ml in acetonitrile – 0.1% (v/v) TFA) was deposited onto the target and left for 5 s before being removed. The residual solution was allowed to air-dry and 0.5 µl sample solution was deposited onto the pre-coated sample spot. Matrix solution of 0.5 µl was added to the deposited sample and allowed to air-dry. The sample was subsequently analyzed in positive-ion linear mode.

### Antimicrobial activity assays

The antimicrobial activity of *S. salivarius* DPC6993 against *F. nucleatum* DSM15643 and *Lactobacillus delbrueckii* subsp. *bulgaricus* DPC5383 was determined by a deferred antagonism assay^62^ in triplicate using three biological replicates. Activity against a range of gram-positive and gram-negative gut bacteria **(Table S1)** was also assessed. FAA (Lab M, Lancashire, UK) and BHI (Difco Laboratories, Detroit, MI, USA) were used for *F. nucleatum* DSM15643 and *S. salivarius* DPC6993, respectively. For the antagonism assay, a fully cultured *S. salivarius* DPC6993 streak plate was overlayed with 0.75% agar seeded with *F. nucleatum* DPC6993 (7.5%). For the spot-on lawn assay, 10 µl of an overnight broth culture of *S. salivarius* DPC6993 was aliquoted onto solid BHI (Difco Laboratories, Detroit, MI, USA) agar and incubated anaerobically at 37°C for 24 h. Subsequently, it was overlayed with FAA (Lab M, Lancashire, UK) seeded with *F. nucleatum* DSM15643 (7.5%) and examined for evidence of inhibition following overnight incubation.

### Donor recruitment for *ex vivo* colon model experiments

Recruitment and enrollment to the study was sanctioned by the Clinical Research Ethics Committee of the Cork Teaching Hospitals (protocol no. APC091). Informed consent was given by all volunteers, which demonstrated their willingness to donate a fecal sample to the study. All donors were healthy adults over the age of 18, and exclusion criteria included no significant acute or chronic coexisting illness; taking a medication that would interfere with the objectives of the study including anti-inflammatory drugs, corticosteroids, laxatives, enemas, antibiotics (within 3 months), anticoagulants, and over-the-counter nonsteroidal analgesics; has been in a recent experimental trial no less than 30 d prior to commencement of this study; and has a malignant disease or any concomitant end-stage organ disease.

### Preparation of a frozen standard fecal inoculum and associated medium

The frozen standard fecal inoculum (FSI) was prepared as previously described^49^ with minor modifications. Briefly, the FSI was prepared using a total of eight donor fecal samples. Potassium phosphate buffer (50 mM; pH 6.8) was used to resuspend the cell biomass resulting in the fecal slurry preparation. Glycerol was added to a final concentration of 25% and the slurry was frozen at −80°C. The medium used was as previously described^63^ and 5% w/v glucose was added to the medium as the carbon source.

### Simulation of the human distal colon

The human distal colon environment was simulated using the micro-Matrix (Applikon Biotechnology, Heertjeslaan 2, 2629 JG Delft, Netherlands) as previously described^7^ with minor modifications. The micro-Matrix is a mini fermentation system capable of simulating the environmental conditions of the human colon. Multiple treatments were applied using separate colon model wells in a micro-Matrix cassette, assigning three wells per treatment group. The fermentation medium and carbon source were mixed in an anaerobic chamber 2 h before commencement of the trial. Then, 480 µL of thawed FSI was inoculated into each well. *S. salivarius* DPC6993 adjusted to a concentration of ~10^9^ CFU/mL to ensure viability and target the minimum recommended probiotic dose^64^ and/or *F. nucleatum* DSM1564 adjusted to a concentration of ~10^6^ CFU/mL to mimic the levels of *F. nucleatum* in human gut microbiomes (relative abundance of <1%)^40,41,65^ was added to each designated well and made up to a final volume of 6 mL using the fermentation medium. Control wells containing just the standardized fecal inoculum and fermentation medium were also included. At Time 0 h (T0), 1 mL was taken from each well of the cassette in the anaerobic chamber to leave a total fermentation volume of 5 mL. The cassette was secured into the micro-Matrix and parts fitted as per the manufacturer’s instructions and fermentation parameters including nitrogen gas (40%), CO_2_ gas, Orbiter (250 RPM), NaOH, pH (6.8), temperature (37°C) and DO (0%) were as previously described.^7^ In addition to thawed SFI and fermentation medium, the cassette was set up with the following colon model treatments: *S. salivarius* DPC6993 with *F. nucleatum* DSM15643; *S. salivarius* DPC6993 only; *F. nucleatum* DSM15643 only and controls.

### DNA extraction from micro-Matrix well samples

At time 0 h (T0), 6 h (T6) and 24 h (T24), 1 mL was taken from each well. Total bacterial metagenomic DNA was extracted from each sample using the Zymo Research ZR fecal DNA kit (Cambridge Biosciences, Cambridge, UK). Each slurry sample was centrifuged at 4000 rpm for 10 min to concentrate the bacterial cells. The supernatant was removed, and cell pellets were frozen at −80°C prior to DNA extraction. The resulting cell biomass was resuspended in lysis buffer and extractions were performed according to the manufacturer’s instructions and quantified using the Qubit 2.0 Fluorometer (Life Technologies, Carlsbad, CA, USA) and the purity checked using the NanoDrop 1000 (ThermoFisher Scientific, Dublin, Ireland).

### Quantitative PCR

Abundances of *F. nucleatum* were determined by real-time PCR based on SYBR-Green I fluorescence.^66^ Absolute quantification of *F. nucleatum* was performed using the Roche LightCycler 96 platform. To quantify *F. nucleatum*, the gene *nusG* was amplified as previously targeted using *F. nucleatum nusG* specific primers.^14^ There is only one copy of *nusG* per *F. nucleatum* genome, and therefore each copy represents a single cell. The primer sequences used were as follows: *nusG* forward primer, 5’-CAACCATTACTTTAACTCTACCATGTTCA-3’; *nusG* reverse primer, 5’- GTTGACTTTACAGAAGGAGATTATGTAAAAATC-3’. BLAST analysis of the primer sequences against the NCBI database confirmed 100% nucleotide identity with the *F. nucleatum nusG* gene sequences and no other matches of concern were evident. Cycling conditions for the qPCR analysis were as follows: 40 cycles of 94°C for 30 s, 55°C for 30 s and 72°C for 1 min. qPCR samples were performed in triplicate. Each qPCR reaction contained 5 μL KAPA SYBR® FAST (2X) (Merck, Product# KK4610), forward and reverse primer (1 μM), PCR grade water and 1 µl of metagenomic DNA in a total reaction volume of 10 µL. Negative controls comprise PCR-grade water replacing metagenomic DNA. For the quantification of the target gene of interest (GOI), standard curves were generated using serial diluted copies of the GOI using a known DNA template.^66^ For quantification of *F. nucleatum* in colon model samples, standard curves were generated using 10^5^ to 10^1^ copies of *nusG*/µL using *F. nucleatum* DSM15643 as a DNA template with an efficiency of 94% and an R-squared value of >0.99. Standard curves were constructed by plotting the quantification cycle (Ct) values versus the log quantity of the target gene in each dilution series. Colon model metagenomic DNA samples were quantified against the standard curve to obtain absolute quantity of *nusG* per μg of DNA. The data analysis was performed using the Roche LightCycler 96 real-time PCR system software.

### MiSeq compositional sequencing and bioinformatic analysis of sequencing data

Metagenomic DNA extracted from colon model samples were prepared for MiSeq compositional sequencing by 16S rRNA amplification of the V3-V4 variable region of the 16S rRNA gene as described by Illumina Inc. Samples were sequenced using the Illumina MiSeq platform in the Teagasc sequencing facility, Moorepark, Fermoy, Ireland. 16S rRNA amplicon analysis was performed using Qiime2 (v. 2018.11.0).^67^ Adapter and primer sequences were removed using cutadapt trim-paired.^68^ The dada2 denoise-paired plugin^69^ was used to trim forward and reverse reads to 283 and 204 bp, respectively, based on quality score visualization by demux summary, and identify amplicon sequence variants (ASVs), and the ASV phylogenetic tree was calculated using phylogeny align-to-tree-mafft-fasttree. The 99% identity 16S rRNA rep set of the Silva 132 database^70^ was downloaded, and the relevant variable regions were isolated from these full-length 16S rRNA sequences using feature-classifier extract-reads^71^ with the primer sequences mentioned above and trimmed to 466 bp based on the median read length of the dataset. These reads were used to train a naïve-Bayes classifier with feature-classifier fit-classifier-naive-Bayes and feature-classifier classify-sklearn^72^ was used to assign taxonomy to the ASVs. Qiime2 artifacts were exported in BIOM format^73^ and used for downstream analysis.

## Statistical analysis

All statistical analysis was computed using R (v3.5.2). The mean quantity of *F. nucleatum* between colon model treatments was compared with the unpaired *t*-test. *p*-values less than 0.05 (*p* < .05) were accepted as a statistically significant difference between the means. The ggplot2 package (v3.2.1) and GraphPad Prism (v9.0) was used to visualize qPCR and 16S rRNA data. Beta and Alpha diversity calculations were completed using the vegan package (v2.5.6). Adonis analysis was implemented using “Adonis” in the package Vegan.^74^ Differences in Alpha diversity metrics were compared using the unpaired *t*-test.

## Supplementary Material

Supplemental Material

## Data Availability

All metagenomic raw reads have been deposited in ENA under accession number PRJEB49010. The *S. salivarius* DPC6993 Whole Genome Shotgun project has been deposited at DDBJ/ENA/GenBank under bioproject number PRJNA819233 and accession JALMLR000000000. The version described in this paper is version JALMLR010000000
