# Supplementary material for: Effect of a bacteriocin-producing Streptococcus salivarius on the pathogen Fusobacterium nucleatum in a model of the human distal colon
Source: Gut Microbes. 2022 Jul 25;14(1):2100203. doi: 10.1080/19490976.2022.2100203 (PMC9318236; doi:10.1080/19490976.2022.2100203)
Supplement: Supplemental Material [file KGMI_A_2100203_SM0846.zip › Revised Supplementary_210390359_Lawrence et al.docx]

**Table S1.** List of bacteria and their culture conditions used in the antimicrobial spectrum analysis

| **Indicator** | **Culture Medium** | **Conditions** | **Source/Reference** |
| --- | --- | --- | --- |
| *Fusobacterium nucleatum* DSM15643 | FAA, 7% Horse blood | Anaerobic | DSMZ Culture Collection |
| *Fusobacterium nucleatum* DSM19507 | FAA, 7% Horse blood | Anaerobic | DSMZ Culture Collection |
| *Fusobacterium nucleatum* DSM19508 | FAA, 7% Horse blood | Anaerobic | DSMZ Culture Collection |
| *Fusobacterium nucleatum* DSM19679 | FAA, 7% Horse blood | Anaerobic | DSMZ Culture Collection |
| *Fusobacterium nucleatum* DSM20482 | FAA, 7% Horse blood | Anaerobic | DSMZ Culture Collection |
| *Fusobacterium nucleatum* D11(BEI) | FAA, 7% Horse blood | Anaerobic | HMP Culture Collection |
| *Fusobacterium nucleatum* CTI-01(BEI) | FAA, 7% Horse blood | Anaerobic | HMP Culture Collection |
| *Fusobacterium varium* DSM19868 | FAA, 7% Horse blood | Anaerobic | DSMZ Culture Collection |
| *Fusobacterium mortiferum* DSM19809 | FAA, 7% Horse blood | Anaerobic | DSMZ Culture Collection |
| *Fusobacterium periodonticum* DSM19545 | FAA, 7% Horse blood | Anaerobic | DSMZ Culture Collection |
| *Lactobacillus bulgaricus* DSM5358 | MRS | Anaerobic | DSMZ Culture Collection |
| *Lactobacillus salivarius* 6005 | MRS | Anaerobic | Teagasc Culture Collection (Casey *et al.*, 2004) |
| *Lactobacillus salivarius* 6488 | MRS | Anaerobic | Teagasc Culture Collection |
| *Lactobacillus salivarius* 6502 | MRS | Anaerobic | Teagasc Culture Collection |
| *Lactobacillus salivarius* UCC118 | MRS | Anaerobic | Teagasc Culture Collection (Riboulet-Bisson *et al.*, 2012) |
| *Enterococcus faecalis* 2.102 | BHI | Aerobic | Faecal sample isolate-Unpublished |
| *Enterococcus faecalis* 7.102 | BHI | Aerobic | Faecal sample isolate-Unpublished |
| *Streptococcus mutans* 110 | BHI | Anaerobic | Faecal sample isolate-Unpublished |
| *Streptococcus agalactiae* 107 | BHI | Aerobic | Faecal sample isolate-Unpublished |
| *Streptococcus salivarius* 3.102 | BHI | Anaerobic | Faecal sample isolate-Unpublished |
| *Bacteroides fragilis 3*_1_12 | BHI+Yeast, Hemin and Vit K | Anaerobic | HMP Culture Collection |
| *Bacteroides intestinalis* DSM17393 | BHI+Yeast, Hemin and Vit K | Anaerobic | DSMZ Culture Collection |
| *Bacteroides fragilis* LMG10263 | BHI+Yeast, Hemin and Vit K | Anaerobic | LMG Culture Collection |
| *Bacteroides thetaiotaomicron* LMG11262 | BHI+Yeast, Hemin and Vit K | Anaerobic | LMG Culture Collection |
| *Bacteroides dorei* DSM17855 | BHI+Yeast, Hemin and Vit K | Anaerobic | DSMZ Culture Collection |
| *Bacteroides sp*. 9_1_42 FAA | BHI+Yeast, Hemin and Vit K | Anaerobic | HMP Culture Collection |
| *Bacteroides uniformis* DSM6597 | BHI+Yeast, Hemin and Vit K | Anaerobic | DSMZ Culture Collection |
| *Bacteroides sp*. 2_1_16 | BHI+Yeast, Hemin and Vit K | Anaerobic | HMP Culture Collection |
| *Clostridium symbiosum* DSM934 | FAA, 7% Horse blood | Anaerobic | DSMZ Culture Collection |
| *Clostridium ramonsum* DSM1402 | FAA, 7% Horse blood | Anaerobic | DSMZ Culture Collection |
| *Clostridioides difficile* DPC6357 | FAA, 7% Horse blood | Anaerobic | Teagasc Culture Collection |
| *Clostridioides difficile* DPC6507 | FAA, 7% Horse blood | Anaerobic | Teagasc Culture Collection |
| *Clostridioides difficile* DPC6506 | FAA, 7% Horse blood | Anaerobic | Teagasc Culture Collection |
| *Clostridioides difficile* DPC6510 | FAA, 7% Horse blood | Anaerobic | Teagasc Culture Collection |
| *Listeria innocua* DPC 3572 | BHI | Aerobic | Teagasc Culture Collection |
| *Listeria innoca* APC1278 | BHI | Aerobic | Teagasc Culture Collection |
| *Salmonella enterica* ATCC BAA-187 | BHI | Anaerobic | Teagasc Culture Collection |
| *Salmonella typhimurium* APC176 | BHI | Anaerobic | Teagasc Culture Collection |
| *Lactobacillus salivarius* DPC6095 | mMRS | Anaerobic | Teagasc Culture Collection |
| *Bifidobacterium breve* DPC6325 | TOS | Anaerobic | Teagasc Culture Collection |
| *Bifidobacterium longum* DPC6316 | TOS | Anaerobic | Teagasc Culture Collection |
| *Streptococcus mutans* APC1076 | BHI | Aerobic | Teagasc Culture Collection |
| *Streptococcus hyointestinalis* APC139 | TSB | Anaerobic | Teagasc Culture Collection |
| *Staphylococcus aureus* DPC5247 | TSB | Aerobic | Teagasc Culture Collection |
| *Staphylococcus chromogens* DPC6012 | TSB | Aerobic | Teagasc Culture Collection |
| *Streptococcus agalactiae* 19247 | BHI | Anaerobic | HMP Culture Collection |
| *Streptococcus agalactiae* SGBS001 | BHI | Anaerobic | HMP Culture Collection |
| *Porphymromonas uenonis* HM130 | BHI | Anaerobic | HMP Culture Collection |
| *Megasphaera micronuciformis* HM1172 | BHI | Anaerobic | HMP Culture Collection |
| *Atopobium parvulum* HM1084 | BHI | Anaerobic | HMP Culture Collection |
| *Gardnerella vaginalis* JCP7275 | BHI | Anaerobic | HMP Culture Collection |
| *Gardnerella vaginalis* 315A | BHI | Anaerobic | HMP Culture Collection |
| *Gardnerella vaginalis* JCP8066 | BHI | Anaerobic | HMP Culture Collection |

FAA, Fastidious Anaerobic Agar; MRS, De man, Rogosa, and Sharpe; BHI, Brain Heart Infusion; mMRS, modified MRS (MRS medium supplemented with 0.05% cysteine); TOS, TOS propionate media; TSB, Tryptic Soy Broth; DSMZ, German Collection of Microorganisms and Cell Culture GmbH; HMP, Human Microbiome Project; LMG, Bacteria Collection Laboratorium voor Microbiologie Universiteit Gent.

**Table S2.** Indicators used in the inhibitory activity spectrum assessment of faecal isolate *S. salivarius* DPC6993 and the degree of inhibition. Zones of inhibition (mm) were measured around single colonies. +: zones of size <5mm, ++: zones of size 5-8mm, +++: zones of size 8-10mm.

| Indicator | Inhibition |
| --- | --- |
| *Fusobacterium nucleatum* DSM15643 | ++ |
| *Fusobacterium nucleatum* DSM19507 | ++ |
| *Fusobacterium nucleatum* DSM19508 | ++ |
| *Fusobacterium nucleatum* DSM19679 | ++ |
| *Fusobacterium nucleatum* DSM20482 | ++ |
| *Fusobacterium nucleatum* D11(BEI) | ++ |
| *Fusobacterium nucleatum* CTI-01(BEI) | ++ |
| *Fusobacterium periodonticum* DSM19545 | + |
| *Lactobacillus delbrueckii* subsp. *bulgaricus* DPC5383 | +++ |
| *Lactobacillus salivarius* DPC6095 | - |
| *Lactobacillus salivarius* DPC6005 | - |
| *Lactobacillus salivarius* DPC6488 | - |
| *Lactobacillus salivarius* DPC6502 | - |
| *Lactobacillus salivarius* UCC118 | - |
| *Bifidobacterium breve* DPC6325 | + |
| *Bifidobacterium longum* DPC6316 | + |
| *Clostridioides difficile* DPC6357 | + |
| *Clostridioides difficile* DPC6506 | + |
| *Clostridium symbiosum* DSM934 | + |
| *Clostridium ramonsum* DSM1402 | - |
| *Clostridioides difficile* DPC6507 | - |
| *Clostridioides difficile* DPC6510 | - |
| *Streptococcus mutans* APC1076 | ++ |
| *Streptococcus hyointestinalis* APC139 | - |
| *Streptococcus agalactiae* 19247 | - |
| *Streptococcus agalactiae* SGBS001 | - |
| *Enterococcus faecalis* 2.102 | - |
| *Enterococcus faecalis* 7.102 | - |
| *Bacteroides fragilis* 3_1_12 | - |
| *Bacteroides intestinalis* DSM17393 | - |
| *Bacteroides fragilis* LMG10263 | - |
| *Bacteroides thetaiotaomicron* LMG11262 | - |
| *Bacteroides dorei* DSM17855 | - |
| *Bacteroides sp*. 9_1_42 FAA | - |
| *Bacteroides uniformis* DSM6597 | - |
| *Bacteroides sp*. 2_1_16 | - |
| *Listeria innocua* DPC3572 | - |
| *Listeria innocua* APC1278 | - |
| *Salmonella enterica* ATCC BAA-187 | - |
| *Salmonella typhimurium* APC176 | - |
| *Staphylococcus aureus* DPC5247 | - |
| *Staphylococcus chromogens* DPC6012 | - |
| *Gardnerella vaginalis* JCP7275 | - |
| *Gardnerella vaginalis* 315A | - |
| *Gardnerella vaginalis* JCP8066 | - |
| *Porphymromonas uenonis* HM130 | - |
| *Megasphaera micronuciformis* HM1172 | - |
| *Atopobium parvulum* HM1084 | - |

| **Colon model**  **treatment** | **Fermentation**  **timepoint**  **(hours)** | ***F. nucleatum* copy**  **number/μl DNA**  **(mean ± SD)** | **Cycle threshold (Ct)**  **(mean ± SD)** |
| --- | --- | --- | --- |
| *S. salivarius* DPC6993 *+*  *F. nucleatum* DSM15643 | 0 | 4452.8±1547.2 | 20.9±0.6 |
|  | 6 | 9243.3±3408.4 | 19.9±0.7 |
|  | 24 | 139.4±39.7 | 25.9±0.5 |
| *S. salivarius* DPC6993 | 0 | 4154.9±1299.5 | 21.0±0.5 |
|  | 6 | 4489.6±1176.7 | 20.9±0.4 |
|  | 24 | 21.3±7.6 | 28.8±0.6 |
| *F. nucleatum* DSM15643 | 0 | 7437.9±1650.7 | 20.1±0.4 |
|  | 6 | 29688.9±4993.9 | 17.9±0.2 |
|  | 24 | 36.9±26.41 | 28.3±1.3 |
| Control | 0 | 3376.9±1814.3 | 21.6±1.3 |
|  | 6 | 2982.5±1474.6 | 21.7±1.0 |
|  | 24 | 2.7±1.1 | 31.9±0.1 |

**Table S3.** Quantification of *F. nucleatum* in colon model wells determined by real time-quantitative polymerase chain reaction (RT-qPCR).

**Table S4.** Relative abundance (%) at bacterial phylum level in colon model wells inoculated with *S. salivarius* DPC6993 + *F. nucleatum* DSM15643, *S. salivarius* DPC6993, *F. nucleatum* DSM15643, and control wells at T0, T6 and T24. Relative abundances are represented by the mean ± SD of 3 colon model wells.

| **Faecal Fermentation Condition** | ***S. salivarius* DPC6993 +**  ***F. nucleatum* DSM15643** | | | ***S. salivarius* DPC6993** | | | ***F. nucleatum* DSM15643** | | | **Control** | | |
| --- | --- | --- | --- | --- | --- | --- | --- | --- | --- | --- | --- | --- |
| **Faecal Fermentation Timepoint** | **T0** | **T6** | **T24** | **T0** | **T6** | **T24** | **T0** | **T6** | **T24** | **T0** | **T6** | **T24** |
| **Phylum** |  |  |  |  |  |  |  |  |  |  |  |  |
| Actinobacteria | 13.35±3.70 | 10.99±1.25 | 9.52±0.54 | 10.95±0.47 | 11.7±1.04 | 10.07±1.45 | 17.3±1.20 | 27.8±5.59 | 29.21±2.29 | 16.92±6.70 | 29.21±4.23 | 29.48±1.8 |
| Bacteroidetes | 1.5±0.25 | 0.61±0.15 | 0.41±0.06 | 2.15±0.57 | 0.67±0.25 | 0.53±0.09 | 1.96±0.16 | 1.09±0.26 | 1.26±0.15 | 1.57±0.34 | 1.67±0.45 | 1.2±0.13 |
| Cyanobacteria | 0.013±0.007 | 0±0 | 0±0 | 0.02±0.03 | 0.004±0.003 | 0.002±0.003 | 0.028±0.01 | 0±0 | 0.002±0.004 | 0.01±0.01 | 0.01±0.01 | 0.01±0.02 |
| Epsilonbacteraeota | 0±0 | 0±0 | 0±0 | 0.1±0.09 | 0±0 | 0±0 | 0±0 | 0±0 | 0±0 | 0±0 | 0±0 | 0±0 |
| Firmicutes | 84.83±3.90 | 85.58±3.07 | 89.13±0.56 | 86.22±0.44 | 84.24±2.69 | 89.18±1.52 | 80.15±1.30 | 45.76±1.58 | 62.96±5.91 | 70.59±24.24 | 43.7±2.88 | 58.2±3.63 |
| Fusobacteria | 0.05±0.04 | 0.03±0.03 | 0±0 | 0.06±0.09 | 0.014±0.02 | 0±0 | 0.13±0.05 | 0.34±0.07 | 0±0 | 0.02±0.04 | 0±0 | 0±0 |
| Lentisphaerae | 0±0 | 0±0 | 0±0 | 0.001±0.002 | 0±0 | 0±0 | 0±0 | 0±0 | 0±0 | 0±0 | 0±0 | 0±0 |
| Patescibacteria | 0.002±0.003 | 0±0 | 0±0 | 0±0 | 0.0006±0.001 | 0±0 | 0±0 | 0±0 | 0±0 | 0.003±0.01 | 0±0 | 0±0 |
| Planctomycetes | 0±0 | 0±0 | 0±0 | 0.002±0.004 | 0±0 | 0±0 | 0±0 | 0±0 | 0±0 | 0±0 | 0±0 | 0±0 |
| Proteobacteria | 0.12±0.06 | 2.77±1.85 | 0.93±0.90 | 0.3±0.21 | 3.34±1.43 | 0.21±0.10 | 0.19±0.05 | 24.99±4.90 | 6.55±7.50 | 10.76±18.40 | 25.39±7.13 | 11.08±1.70 |
| Spirochaetes | 0±0 | 0±0 | 0±0 | 0.001±0.002 | 0±0 | 0±0 | 0±0 | 0±0 | 0±0 | 0±0 | 0±0 | 0±0 |
| Synergistetes | 0±0 | 0±0 | 0±0 | 0.01±0.02 | 0±0 | 0±0 | 0±0 | 0±0 | 0±0 | 0±0 | 0±0 | 0±0 |
| Tenericutes | 0.04±0.01 | 0.07±0.01 | 0.004±0.01 | 0.03±0.01 | 0.01±0.01 | 0.01±0.01 | 0.05±0.02 | 0.01±0.01 | 0.01±0.01 | 0.05±0.03 | 0.01±0.002 | 0.02±0.01 |
| Verrucomicrobia | 0.11±0.04 | 0.01±0.01 | 0.001±0.002 | 0.16±0.07 | 0.02±0.01 | 0.01±0.01 | 0.20±0.04 | 0±0 | 0.01±0.01 | 0.080±0.06 | 0.02±0.01 | 0.01±0.01 |

**Table S5.** Relative abundance (%) at bacterial genera level of top 20 most represented genera in colon model wells inoculated with *S. salivarius* DPC6993 + *F. nucleatum* DSM15643, *S. salivarius* DPC6993, *F. nucleatum* DSM15643, and control wells at T0, T6 and T24. Relative abundances are represented by the mean ± SD of 3 faecal fermentation wells.

| **Faecal Fermentation Condition** | ***S. salivarius* DPC6993 +**  ***F. nucleatum* DSM15643** | | | ***S. salivarius* DPC6993** | | | ***F. nucleatum* DSM15643** | | | **Control** | | |
| --- | --- | --- | --- | --- | --- | --- | --- | --- | --- | --- | --- | --- |
| **Faecal Fermentation Timepoint** | **T0** | **T6** | **T24** | **T0** | **T6** | **T24** | **T0** | **T6** | **T24** | **T0** | **T6** | **T24** |
| **Genus** |  |  |  |  |  |  |  |  |  |  |  |  |
| *Bifidobacterium* | 9.43±2.36 | 9.31±1.03 | 8.14±0.37 | 7.44±0.45 | 9.78±1.16 | 8.71±0.98 | 11.57±0.84 | 25.21±5.11 | 25.69±2.10 | 13.37±7.24 | 26.32±3.97 | 26.77±1.63 |
| *Collinsella* | 3.24±1.11 | 1.44±0.28 | 0.89±0.03 | 2.87±0.17 | 1.69±0.18 | 1.13±0.54 | 4.77±0.54 | 2.38±0.84 | 3.08±0.69 | 3.01±1.03 | 2.64±0.28 | 2.20±0.47 |
| *Bacteroides* | 0.64±0.13 | 0.38±0.09 | 0.35±0.05 | 1.06±0.44 | 0.46±0.14 | 0.45±0.09 | 0.84±0.09 | 0.74±0.12 | 1.11±0.17 | 0.78±0.13 | 1.20±0.48 | 1.08±0.05 |
| *Globicatella* | 0±0 | 0±0 | 1.61±0.77 | 0±0 | 0±0 | 2.3±0.73 | 0±0 | 0±0 | 2.25±0.94 | 0±0 | 0±0 | 3.04±0.47 |
| *Enterococcus* | 0±0 | 0.04±0.02 | 3.10±0.36 | 0.01±0.02 | 0.02±0.02 | 2.87±1.57 | 0±0 | 0.21±0.06 | 3.21±1.99 | 0.06±0.11 | 0.18±0.06 | 3.73±2.50 |
| *Streptococcus* | 26.28±21.28 | 70.92±4.23 | 70.15±1.17 | 31.28±0.81 | 68.81±3.41 | 66.9±3.11 | 1.19±0.09 | 24.08±3.02 | 24.43±8.60 | 19.001±17.90 | 18.71±1.44 | 26.02±2.5 |
| *Christensenellaceae R-7 group* | 1.82±0.66 | 0.32±0.03 | 0.12±0.02 | 1.73±0.04 | 0.31±0.02 | 0.21±0.03 | 2.59±0.35 | 0.40±0.11 | 0.25±0.13 | 1.48±1.02 | 0.46±0.06 | 0.19±0.05 |
| *Clostridium sensu stricto 1* | 1.40±0.44 | 0.59±10.5 | 0.40±0.11 | 1.21±0.12 | 0.6±0.23 | 0.72±0.33 | 2.02±0.16 | 1.24±0.63 | 1.28±0.45 | 1.33±0.52 | 0.98±0.004 | 0.96±0.34 |
| *Peptoniphilus* | 0.0009±0.0 | 0±0 | 4.84±0.59 | 0±0 | 0.001±0.002 | 5.49±1.07 | 0.002±0.004 | 0±0 | 1.8±2.04 | 0±0 | 0.004±1.13 | 2.92±2.56 |
| *Anaerostipes* | 0.51±0.21 | 0.19±0.12 | 0.06±0.01 | 0.42±0.05 | 0.22±0.12 | 0.12±0.08 | 0.82±0.07 | 1.38±0.70 | 5.27±7.85 | 0.66±0.32 | 2.02±0.45 | 0.79±0.12 |
| *Blautia* | 7.17±3.14 | 2.15±0.43 | 0.40±0.09 | 8.79±0.10 | 2.48±0.17 | 0.78±0.32 | 10.47±1.90 | 2.67±0.50 | 2.25±2.35 | 7.85±5.1 | 2.42±0.13 | 0.91±0.08 |
| *Fusicatenibacter* | 2.04±0.88 | 0.39±0.07 | 0.13±0.02 | 2.15±0.13 | 0.55±0.06 | 0.28±0.07 | 2.65±0.55 | 0.43±0.04 | 0.57±0.40 | 1.82±1.21 | 0.38±0.08 | 0.38±0.03 |
| *[Eubacterium] eligens group* | 1.55±0.46 | 0.29±0.06 | 0.15±0.06 | 1.22±0.07 | 0.32±0.04 | 0.19±0.04 | 2.02±0.23 | 0.25±0.02 | 0.21±0.03 | 1.17±0.74 | 0.36±0.08 | 0.14±0.20 |
| *Romboutsia* | 3.07±0.83 | 0.77±0.16 | 0.71±0.08 | 2.63±0.22 | 0.91±0.10 | 0.92±0.14 | 4.13±0.08 | 0.78±0.14 | 1.22±0.27 | 2.50±1.53 | 0.82±0.34 | 0.97±0.50 |
| *Faecalibacterium* | 9.67±1.91 | 0.36±0.10 | 0.05±0.01 | 8.83±0.28 | 0.43±0.06 | 0.08±0.02 | 12.34±0.94 | 0.83±0.25 | 0.46±0.45 | 6.66±5.51 | 0.93±0.20 | 0.13±0.02 |
| *Ruminococcus 2* | 4.84±1.56 | 0.87±0.25 | 0.50±0.13 | 3.87±0.04 | 0.96±0.05 | 0.63±0.15 | 6.36±0.56 | 1.02±0.06 | 1.71±0.61 | 4.12±2.47 | 1.5±0.36 | 1.89±0.13 |
| *Subdoligranulum* | 6.96±1.17 | 1.66±0.33 | 1.01±0.05 | 6.14±0.09 | 1.96±0.17 | 1.44±0.29 | 8.27±0.07 | 1.62±0.19 | 1.77±0.43 | 5.18±3.06 | 1.66±0.20 | 1.37±0.39 |
| *Erysipelotrichaceae UCG-003* | 1.13±0.39 | 1.86±0.34 | 1.06±0.20 | 0.89±0.04 | 1.26±0.26 | 0.87±0.06 | 1.58±0.14 | 3.80±0.30 | 4.11±0.14 | 2.93±2.75 | 4.70±0.65 | 4.69±0.80 |
| *Phascolarctobacterium* | 0.27±0.10 | 0.47±0.08 | 0.43±0.02 | 0.27±0.03 | 0.60±0.02 | 0.51±0.04 | 0.44±0.14 | 0.68±0.30 | 1.45±0.45 | 0.33±0.14 | 0.82±0.37 | 1.61±0.60 |
| *Escherichia-Shigella* | 0.08±0.04 | 3.47±0.55 | 0.83±0.89 | 0.23±0.18 | 3.22±1.49 | 0.08±0.06 | 0.13±0.01 | 24.97±4.93 | 6.14±7.59 | 10.73±18.42 | 25.33±7.17 | 10.8±1.44 |

**
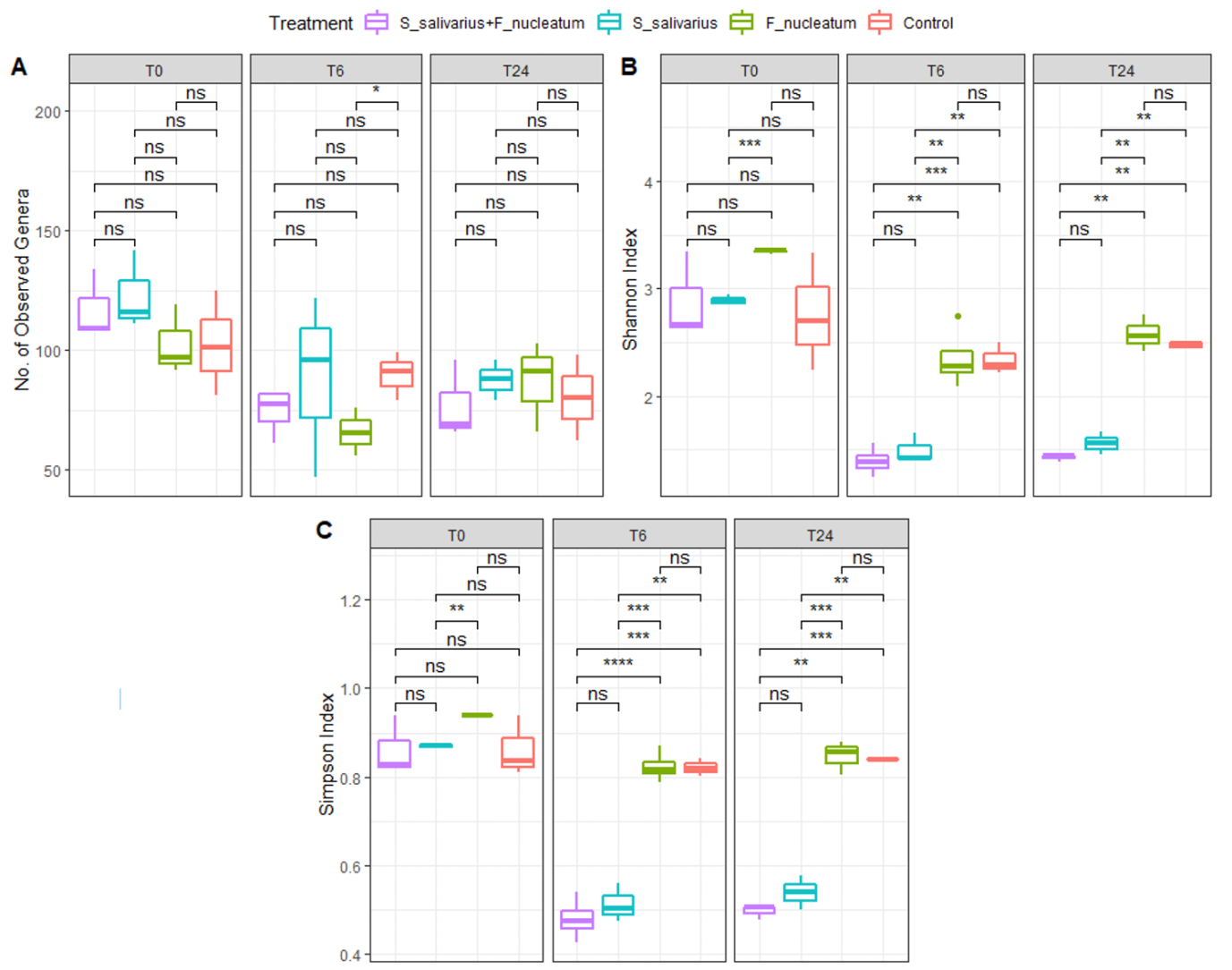
**

**Figure S1. Comparison of alpha diversity metrics.** Number of Observed Genera (A), Shannon Index (B) and Simpson Index (C) were calculated between colon model treatments within timepoints T0, T6 and T24. The microbial richness and diversity were compared by unpaired t-test. ns, not significant; *, P < 0.05; **, P < 0.01; ***, P < 0.001; ****, P < 0.0001.
