# Supplementary figures and images for: Effect of a bacteriocin-producing Streptococcus salivarius on the pathogen Fusobacterium nucleatum in a model of the human distal colon
Source: Gut Microbes. 2022 Jul 25;14(1):2100203. doi: 10.1080/19490976.2022.2100203 (PMC9318236; doi:10.1080/19490976.2022.2100203)

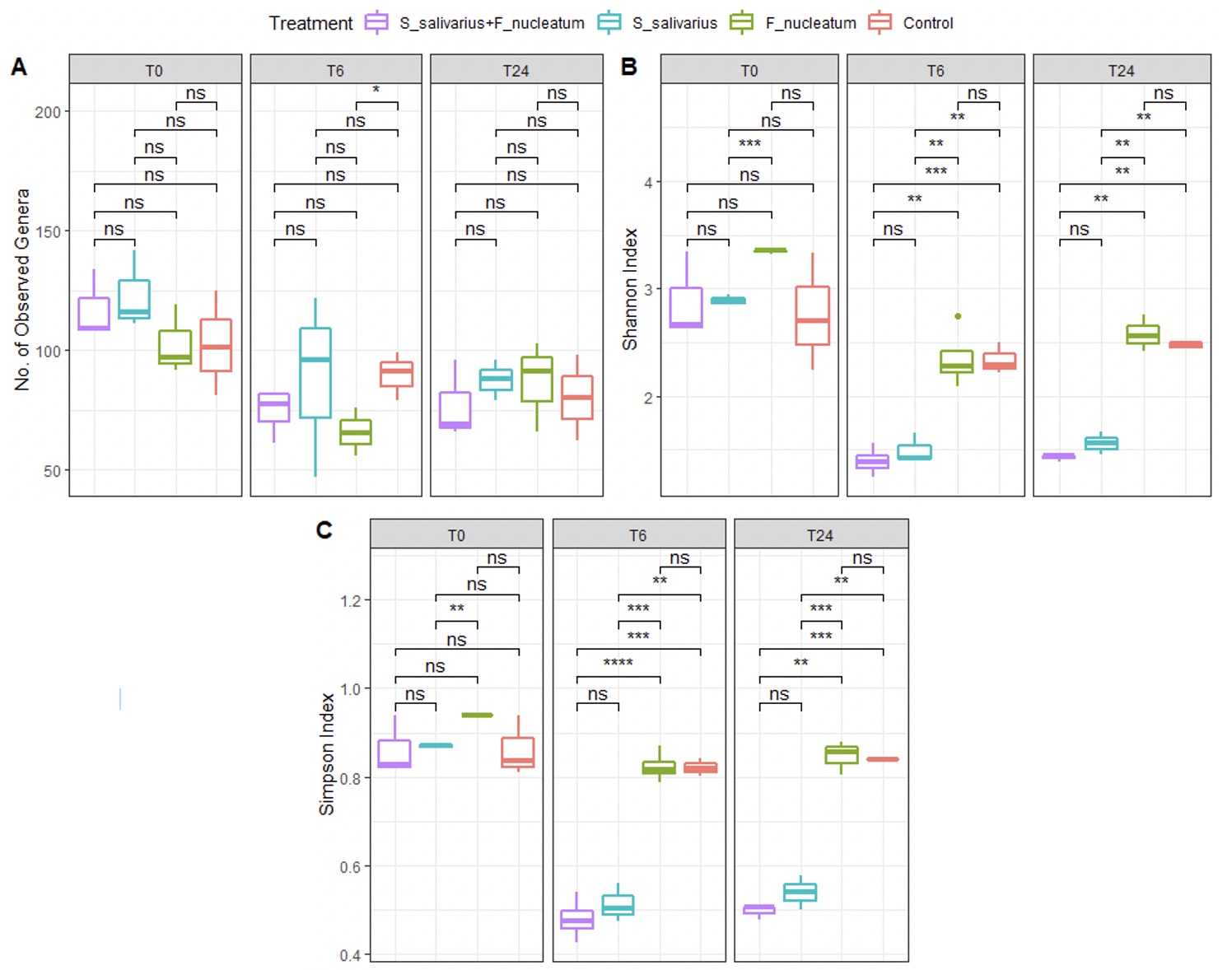

Supplement: Supplemental Material [file KGMI_A_2100203_SM0846.zip › Figure_S1.jpg]
